# Oxygen nanocarrier broke the hypoxia trap of solid tumors and rescued transfection efficiency for gene therapy

**DOI:** 10.1186/s12951-021-01144-4

**Published:** 2021-12-18

**Authors:** Bing Qin, Mengshi Jiang, Xiang Li, Yingying Shi, Junlei Zhang, Zhenyu Luo, Lihua Luo, Yichao Lu, Xu Liu, Sijie Wang, Yongzhong Du, Yunqing Qiu, Yan Lou, Jian You

**Affiliations:** 1 College of Pharmaceutical Sciences, Zhejiang University, 866 Yuhangtang Road, Hangzhou, 310058 Zhejiang People’s Republic of China; 2 Zhejiang Provincial Key Laboratory for Drug Evaluation and Clinical Research, Department of Clinical Pharmacy, The First Affiliated Hospital, Zhejiang University School of Medicine, Zhejiang, People’s Republic of China

**Keywords:** Hypoxia, PFOB, Oxygen delivery, Gene therapy

## Abstract

**Background:**

Gene therapy shows great promise for a broad array of diseases. However, we found that hypoxic tumor microenvironment (TME) exerted significant inhibitory effects on transfection efficiency of a variety of gene vectors (such as Lipo 2000 and PEI) in an oxygen-dependent manner. Solid tumors inevitably resulted in acute hypoxic areas due to the rapid proliferation of tumor cells and the aberrant structure of blood vessels. Thus, the hypoxic TME severely limited the efficiency and application of gene therapy.

**Methods:**

In our previous study, we constructed endoplasmic reticulum-targeted cationic liposomes, PAR-Lipo, which could effectively deliver genes and ensure high transfection efficiency under normoxia. Unsatisfactorily, the transfection efficiency of PAR-Lipo was rather poor under hypoxia. We believed that reoxygenation was the most direct and effective means to rescue the low transfection under hypoxia. Hence, we fabricated liposomes modified with perfluorooctyl bromide (PFOB@Lipo) to load oxygen and deliver it to tumor sites, which effectively alleviated the hypoxic nature of tumor. Then PAR-Lipo were applied to mediate high-efficiency delivery of tumor suppressor gene pTP53 to inhibit tumor progression.

**Results:**

The results showed that such staged strategy augmented the expression of P53 protein in tumors and extremely suppressed tumor growth.

**Conclusion:**

This work was the first attempt to utilize an oxygen nanocarrier to assist the therapeutic effect of gene therapy under hypoxia, providing a new reference for gene therapy in malignant tumors.

**Graphical Abstarct:**

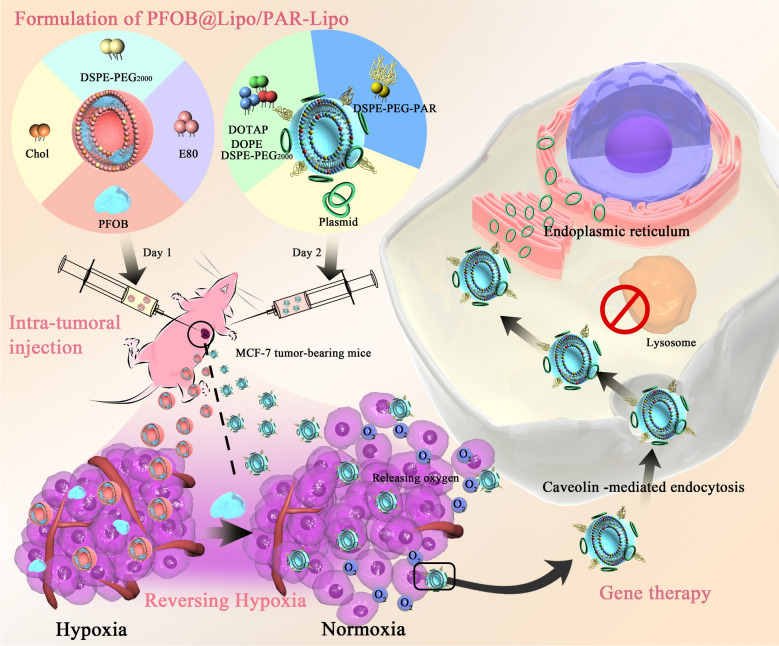

## Introduction

Gene therapy aims to introduce therapeutic nucleic acids [(deoxyribonucleic acid (DNA), ribonucleic acid (RNA), or oligonucleotides)] into target cells to correct or compensate disease caused by genetic defects and anomalies, so as to treat or prevent a wide range of disease [1]. So far, gene therapy products have approved by the United States Food and Drug Administration (US FDA) include Patisiran for the treatment of polyneuropathy in people with hereditary transthyretin-mediated amyloidosis, and Zolgensma for the treatment of spinal muscular atrophy, etc [2]. Simultaneously, COVID-19 vaccines are being investigated by many countries widely. The approval of the Pfizer–BioTech vaccine was regarded as a key milestone for the mRNA-based vaccine platform, since it had never been approved for use in vaccines before [3]. These implied that gene therapy has undoubtedly received extensive attention and become a powerful treatment. The therapeutic effect of gene therapy largely depends on the capacity of gene vectors to deliver exogenous genes and the efficiency of gene transfection in target cells [4]. Most of the related work focused on optimizing gene delivery systems to enhance the efficiency of gene therapy through effective encapsulation, enhanced cellular uptake of target cells, endosomal escape and promoted nuclear transport [5, 6]. These methods only achieved highly efficient gene transfection in vitro, the therapeutic effect in vivo was still far from satisfactory [7]. The most striking example is the tumor gene therapy failed to achieve ideal results, primarily due to the highly complex TME. Regrettably, few studies paid attention to the status of TME on the efficiency in gene therapy.

Malignant cellular proliferation and aberrant neovascularization cause tumor hypoxia inevitably, which is a common characteristic in rapidly growing solid tumors [8]. Notably, the adaptation of progressing tumors to hypoxia not only accelerates tumor invasion and metastasis but inhibits T lymphocytes functions and antigen presentation capacity of dendritic cells (DCs), thereby strongly enhancing the immunosuppressive microenvironment and greatly reducing the efficacy of various treatments, including gene therapy [9–12]. There are two major obstacles to tumor gene therapy in the clinic that needed to be addressed. First, the delivery of genes to hypoxic regions is challenging since such regions are distant from blood vessels and have increased efflux transporters [13]. Second, gene expression may be extremely restrained by hypoxic conditions [14–19]. Oxygen plays a vital role in generating ATP to maintain a productive rate of gene expression in normoxia. Compared to DNA replication or transcription, the translation of mRNA is the most energy-consuming activity within cells [20, 21]. In hypoxia, the rate of the gene translation is dramatically slowed and impaired due to limited ATP availability [22]. Therefore, alleviating tumor hypoxia would significantly enhance the effect of gene therapy in many aspects.

Direct oxygen delivery with high-oxygen-affiliative materials is one of the most promising methods to increase tumor oxygenation for various cancer therapy [23]. Perfluorocarbons (PFCs), a kind of chemically inactive and nontoxic compound, have been widely utilized as an innovative material in artificial oxygen vectors owing to their high oxygen-carrying capacity and superior biocompatibility [24–27]. PFCs can dissolve a large amount of oxygen through the gaseous diffusion process, and transport oxygen through passive diffusion in oxygen-deficient areas [28]. The oxygen solubility of PFCs is proportional to the partial pressure of the oxygen, so oxygen can be released from it under hypoxia quickly. We previously designed an oxygen­enriching perfluorocarbon nanosystem, PFC@Lipo, to overcome hypoxia and enhance photothermal-chemotherapy and immunotherapy [29, 30]. Nevertheless, to date, few if any PFCs nanocarriers with an oxygen-delivering ability to reverse tumor hypoxia and sensitize gene therapy have been reported.

**Scheme 1:**
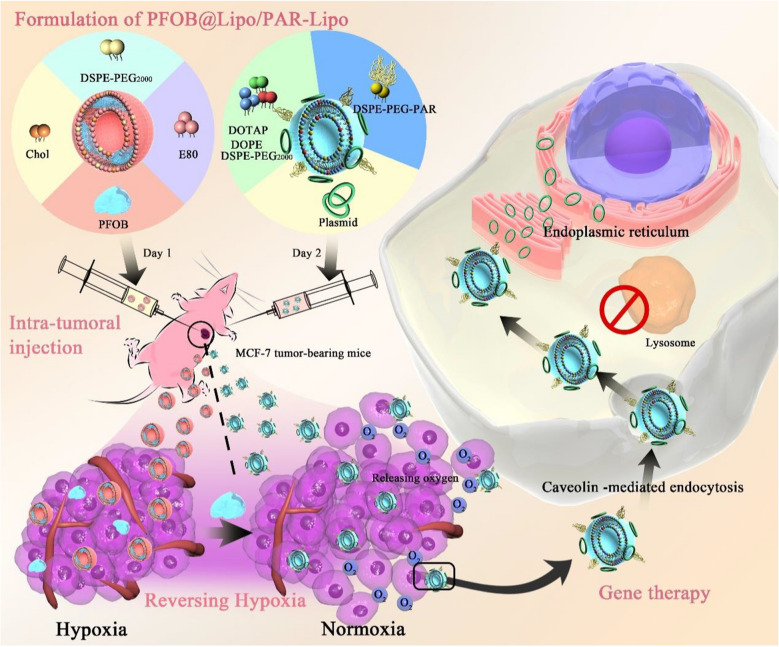
Schematic illustration of O_2_@PL enhanced oxygen levels and reversal of hypoxia-induced transfection efficiency suppression

Accordingly, we proposed here a staged therapeutic strategy. Firstly, to alleviate hypoxia, the PFOB liposomes loaded oxygen (expressed as O_2_@PL) were applied to deliver oxygen to the hypoxic tumor sites. Then we used endoplasmic reticulum targeting cationic liposomes, PAR-Lipo, to efficiently delivery tumor suppressor gene pTP53 [31]. In our previous study, PAR-Lipo exhibited excellent gene delivery and transfection capacity under normoxia [32, 33]. In this work, we first examined the transfection efficiency of various commonly used gene vectors under normoxia and hypoxia, and further investigated the synergistic effect of oxygen with gene therapy in suppressing tumor growth (Scheme 1). Obtained results indicated that our strategy effectively broke the restriction of hypoxia on gene therapy and greatly delayed tumor growth. To the best of our knowledge, the present work is the first attempt to utilize oxygen to rescue the rare expression of tumor suppressor genes, which may be beneficial to the further clinical application of gene therapy in tumor treatment.

## Results

### Characteristics of PFOB@Lipo and capacity to relieve hypoxia in vitro

**Fig. 1:**
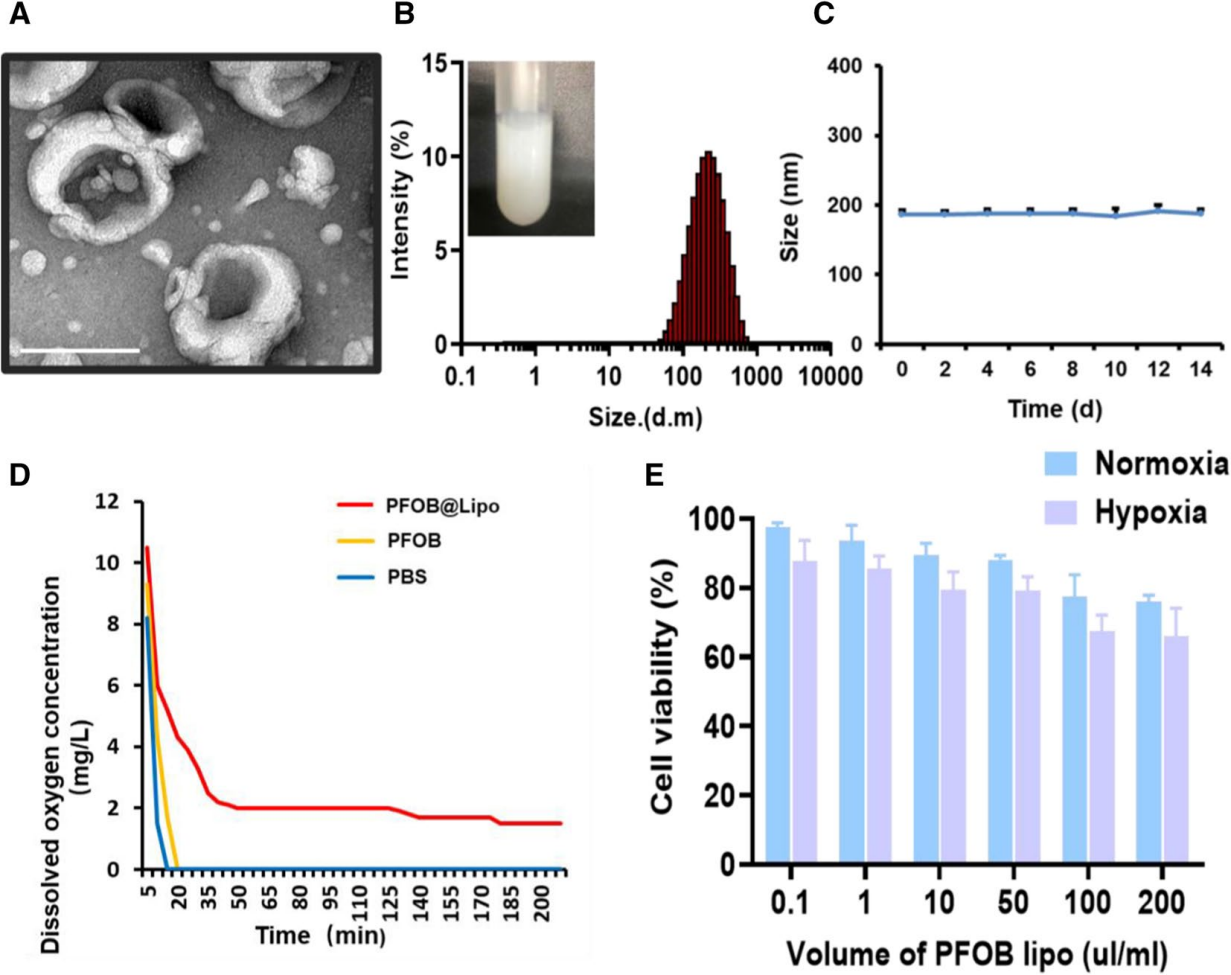
Characterization and cell cytotoxicity of PFOB@Lipo. **A** TEM image of PFOB@Lipo. Scale bar, 200 nm. **B** Particle size of the PFOB@Lipo determined by DLS. **C** Change in particle size of PFOB@Lipo stored at 4 °C for 14 days. **D** Dissolved oxygen measurement results of PFOB@Lipo, PFOB and PBS after oxygenation for 15 min. **E** Cell viability of MCF-7 cells incubated with different concentration of PFOB@Lipo. All error bars are expressed as ± SD, n = 5

In this study, we prepared PFOB@Lipo by the thin film dispersion method. As seen in the transmission electron microscopy (TEM) image, PFOB@Lipo were rather uniform and spherical (Fig. 1A), with an average diameter of 190 nm determined by dynamic light scattering (DLS) (Fig. 1B), and this value did not change after 14 days of storage, suggesting the long-term stability of the PFOB@Lipo in phosphate-buffered saline (PBS) (Fig. 1C). Owing to the superior oxygen dissolving ability of PFOB, we speculated that PFOB@Lipo should have the potential to deliver oxygen. To determine the oxygen-storing capacity and oxygen release behavior, different samples including PFOB@Lipo, PFOB and PBS were pre-oxygenated, and respectively added to anaerobic culture medium. Oxygen release process was monitored by oxygen electrode. Obviously, PFOB@Lipo had the highest increase in oxygen content and the slowest oxygen release (Fig. 1D). Such phenomenon may be beneficial for sustained oxygen release in hypoxic tumor sites. 48 h cytotoxicity was measured by the MTT assay. PFOB@Lipo had no direct killing effect on tumor cells under either normoxic or hypoxic conditions (Fig. 1E).

### Low transfection efficiency was caused by translational repression

**Fig. 2:**
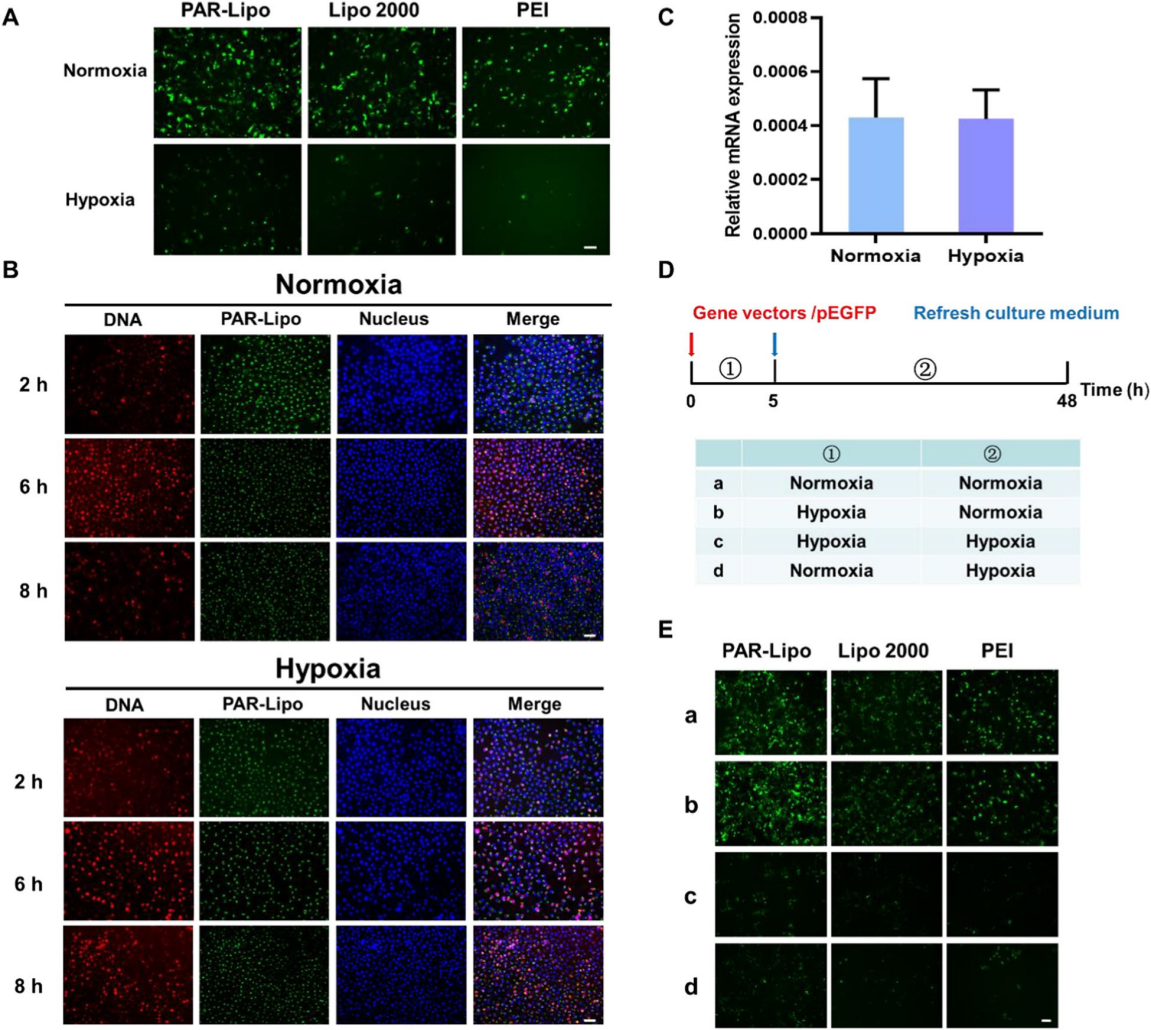
GFP expression, cellular uptake, and mRNA expression of PAR-Lipo/plasmid in MCF-7 cells. **A** GFP expression in MCF-7 cells after incubation with PAR-Lipo/pEGFP (5:1, w/w), Lipo 2000/pEGFP (1.5:1, w/w) and PEI/pEGFP (2:1, w/w) complexes under hypoxia or nomoxia. Scale bars, 100 μm. **B** Cellular uptake of PAR-Lipo/Cy-3 DNA by MCF-7 cells under normoxia or hypoxia. Scale bar, 100 μm. **C** Relative mRNA expression of EGFP under hypoxia or nomoxia. **D** Schematic diagram of transfection under different conditions. **E** GFP expression in MCF-7 cells after treatment as described in **D**. All error bars are expressed as ± SD, n = 4

To evaluate the transfection efficiency of different gene vectors in MCF-7 cells, we chose the enhanced green fluorescent protein plasmids (pEGFP) as a report gene. Surprisingly, the transfection efficiency in PAR-Lipo, Lipo 2000 and PEI groups were all extremely reduced under hypoxic conditions (Fig. 2A). In order to explore the underlying mechanisms accounting for the sharply decreased transfection efficiency under hypoxia, we investigated the following three aspects that were reported to be closely related to transfection efficiency: the uptake of transfection complexes, the transcription of gene and the mRNA translation. Firstly, the time-dependent (2, 6, 8 h) cellular uptake of the complexes was monitored by co-incubating MCF-7 cells with DiD-labeled complexes. No matter in normoxia or hypoxia, the intracellular fluorescence of complexes both increased at 2 h, which was distributed in almost every cell at 6 h, suggesting that the hypoxia did not disturb the cellular internalization of the complexes (Fig. 2B). Then, the mRNA level in cells transfected with PAR-Lipo/pEGFP complex under different conditions for 24 h was explored. Results showed that there was no difference in GFP mRNA levels under hypoxia and normoxia, suggesting that hypoxia did not suppress the transcription of exogenous genes (Fig. 2C). Following the steps shown in Fig. 2D, we categorized four groups based on the time of gene expression and then identified the expression of GFP under different conditions. Interestingly, it was found that as long as protein expression was performed under normoxia, the transfection efficiency could be greatly restored even when the transfection complexes were added under hypoxia (Fig. 2E). Based on the above observations, we believed that such reduced transfection efficiency under hypoxia might lie in the dramatically repressed protein translation process, rather than the cellular uptake and transcription behaviors. It was reported that the addition of glucose or reoxygenation restored translation in hypoxic PC3 cells. Furthermore, reoxygenating the hypoxic PC3 cells by returning them to normoxic conditions for 1 h completely restored translation, even without glucose [17]. Therefore, oxygen plays a vital role in gene translation. Our study provided experimental basis for the co-delivery of oxygen and gene in tumor gene therapy.

### Co-delivery of oxygen enhanced the gene delivery efficiency in vitro and in vivo

**Fig. 3:**
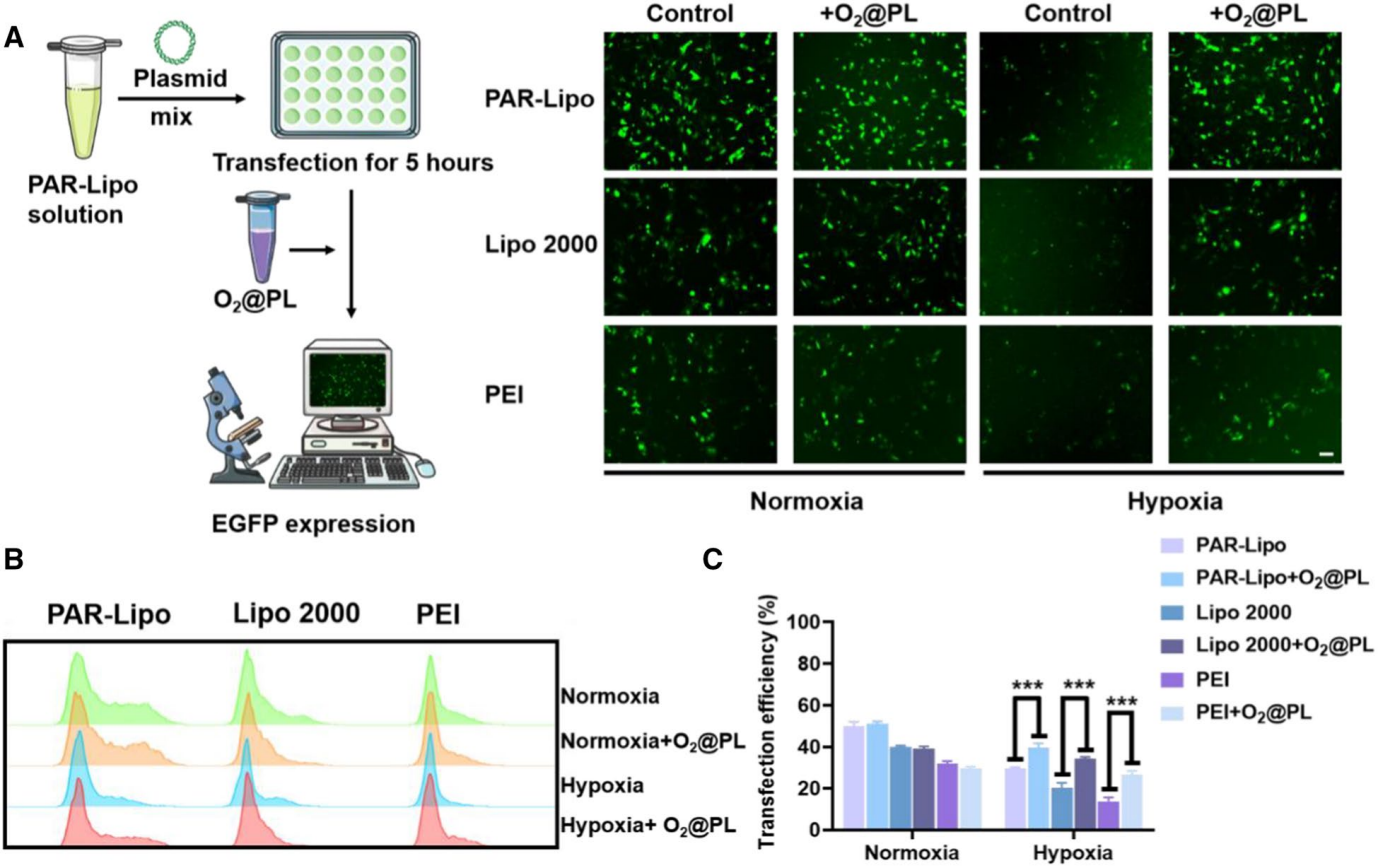
Co-delivery of oxygen enhanced the gene delivery efficiency in vitro. **A** GFP expression in MCF-7 cells after incubation with PAR-Lipo/pEGFP, Lipo 2000/pEGFP and PEI/pEGFP complexes with/without O_2_@PL under hypoxia or nomoxia conditions. Scale bars, 100 μm. **B** Positive rate of GFP expression was determined by flow cytometry after 24 h transfection. **C** Quantitative results of **B**. All error bars are expressed as ± SD, n = 5

High gene delivery capacity is the basis of effective gene therapy. To evaluate the transfection efficiency of different gene vectors after co-delivery of oxygen, we used pEGFP with different gene vectors to transfect MCF-7 cells. To maximize transfection efficiency under hypoxia, we added O_2_@PL 5 h after the addition of transfection complexes in subsequent experiments. Figure 3A demonstrated that the hypoxic environment obviously limited the gene expression, but the addition of O_2_@PL reversed the above phenomenon, making the expression of GFP in each group under hypoxia approach those under normoxia. The percentage of GFP^+^ cells was then quantified using flow cytometry (Fig. 3B, C). For the samples treated with different gene vectors/pGFP plus O_2_@PL, the percentage of GFP-positive cells elevated due to the addition of the O_2_. The PAR-Lipo group increased from 27.8% to 39.8%, the Lipo 2000 group increased from 20.2% to 34.3%, and the PEI group improved from 13.8% to 26.6%, respectively. However, there was no obvious difference after addition of O_2_@PL in each group under normoxia. The synergistic effect of oxygen-carrier PFOB@Lipo made the transfection efficiency of PAR-Lipo under hypoxia beat the commercial cationic liposome Lipo 2000 and gold standard PEI under normoxia. In sum, the delivery of oxygen markedly rescued the low transfection efficiency mediated by hypoxia.

**Fig. 4:**
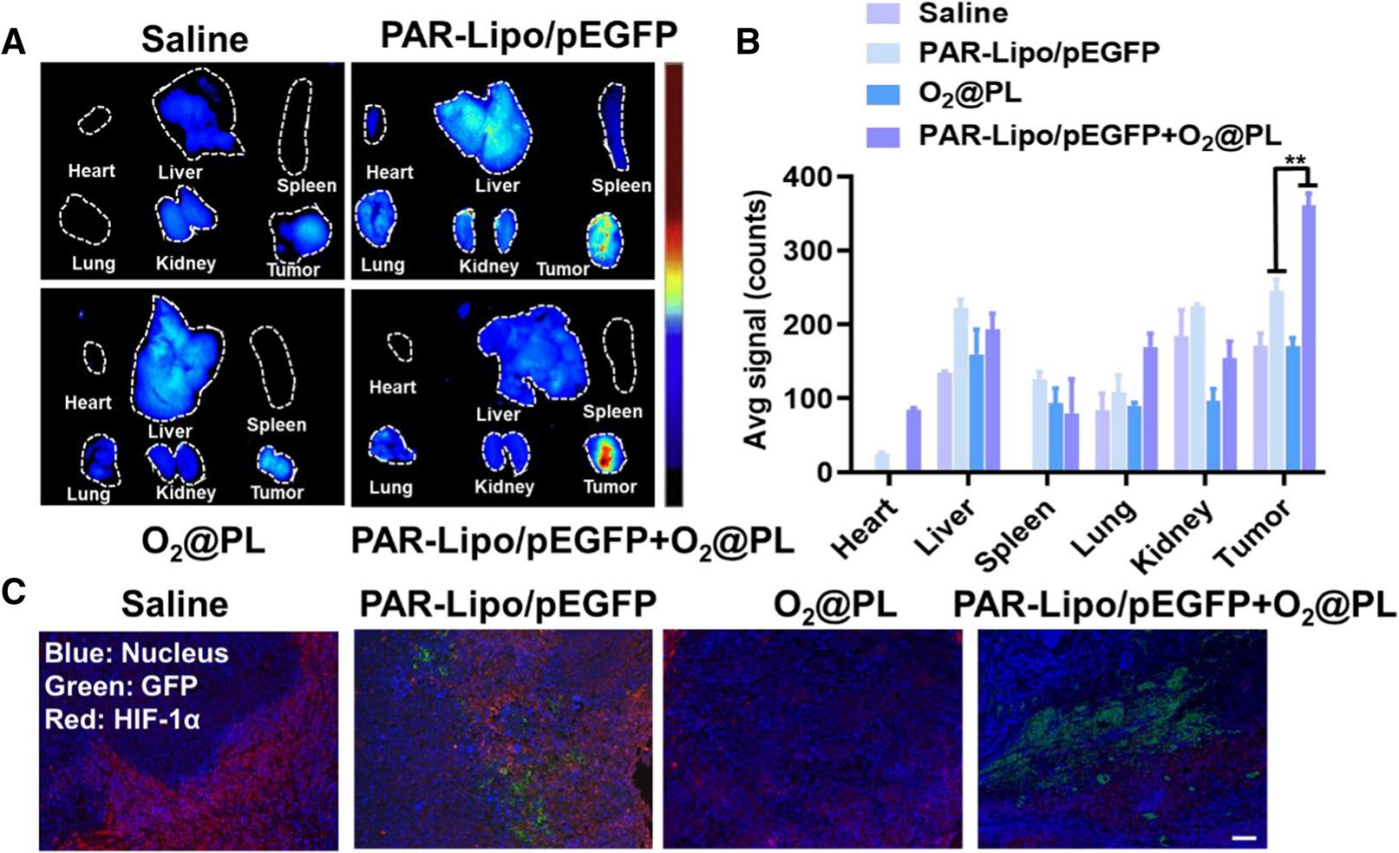
Oxygen delivery of oxygen enhanced the gene delivery efficiency in vivo. **A** GFP expression in mice bearing MCF-7 tumors after intratumoral injection with Saline, PAR-Lipo/pEGFP, O_2_@PL and PAR-Lipo/pEGFP + O_2_@PL. **B** Quantitative results of fluorescence intensity in **A**. **C** Immunofluorescence staining of GFP (green) and HIF-1α(red) after different treatment in vivo. Scale bars, 100 μm. All error bars are expressed as ± SD, n = 5

Solid tumors were obviously heterogeneous and their microenvironment was highly complex, we further investigated whether the delivery of O_2_@PL could effectively alleviate hypoxia in vivo to reproduce the results in vitro. Mice bearing MCF-7 tumors were intratumorally injected with O_2_@PL for two consecutive days to alleviate hypoxia and promote penetration of transfection complexes first, and then intratumorally injected with PAR-Lipo/pEGFP once. The ex vivo fluorescence and semi-quantitative analysis of various tissues at 24 h illustrated that the highest GFP signal were found in the tumor of PAR-Lipo/pEGFP + O_2_@PL treated group (Fig. 4A, B). The tumors were sliced to observe the expression of GFP more clearly. Compared with the control group, the expression of HIF-1α (red) was dramatically reduced, while the expression of GFP was strongly increased in PAR-Lipo/pEGFP + O_2_@PL group (Fig. 4C). In summary, the addition of O_2_@PL combined with PAR-Lipo/pEGFP presented an enhanced gene transfection efficacy as well as protein expression.

### P53 gene transfection study after alleviating hypoxia in vitro

**Fig. 5:**
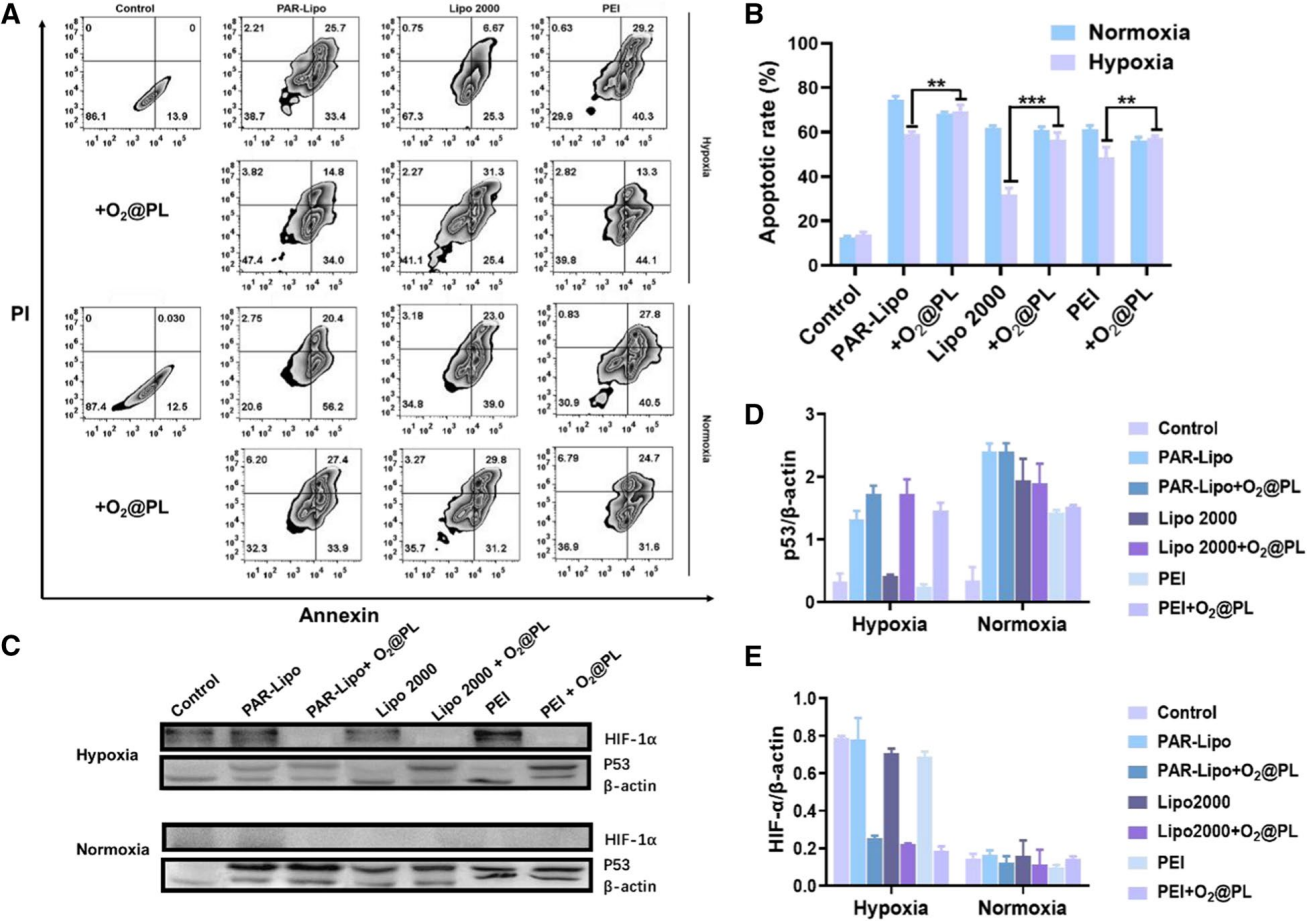
The impact of O_2_@PL on the transfection of pTP53. **A** Annexin V-FITC/PI co-staining assay of MCF-7 cells after various treatments determined by flow cytometry. **B** Quantitative results of apoptosis rates in (**A**). **C** Western blot analysis of the P53 and HIF-1α protein expression levels in MCF-7 cells after various treatments. The proportion of P53 (**D**) and HIF-1α (**E**) relative to that in the control group are presented. All error bars are expressed as ± SD, n = 3

The P53 gene, a tumor suppressor gene that can induce tumor cell cycle arrest, senescence and apoptosis, was used as a therapeutic plasmid for further antitumor investigation. The results revealed that the necrosis and apoptosis rates of MCF-7 cells in the PAR-Lipo/pTP53 group were higher than those in the Lipo 2000/pTP53 and PEI/pTP53 groups under normoxia (Fig. 5A, B). However, regardless of which gene vector was used to deliver pTP53, its ability to induce apoptosis was enormously reduced under hypoxia, but could be greatly saved by the addition of O_2_@PL. Then, the protein expression of P53 and HIF-1α in MCF-7 cells was explored. The higher P53 protein levels and lower HIF-1α levels in Fig. 5C–E indicated that the alleviated hypoxia achieved by O_2_@PL promoted the expression of tumor suppressor gene, mediating a remarkably enhanced therapeutic efficacy against cancer cells.

### O_2_@PL alleviated hypoxia and enhanced gene therapeutic effect in mouse breast cancer.

**Fig. 6:**
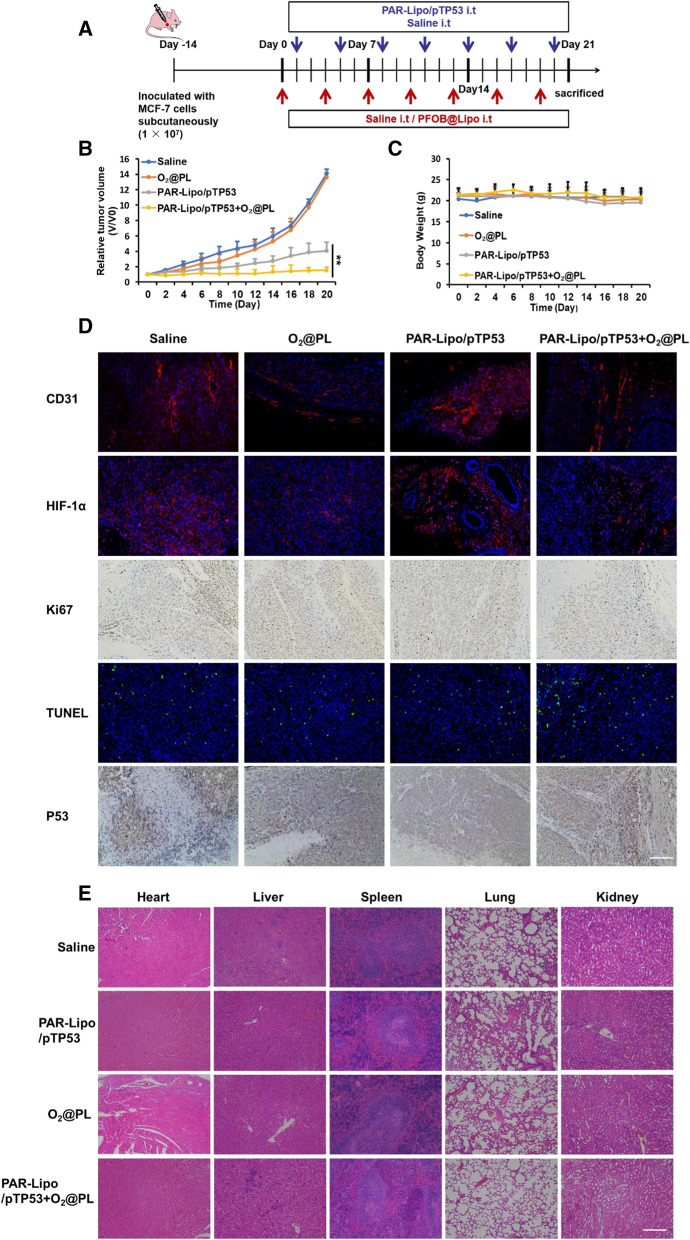
Synergistic anti-tumor effect of O_2_@PL and PAR-Lipo/pTP53 on MCF-7 tumors. **A** Schematic diagram of administration method for mice bearing MCF-7 tumors (n = 6). The relative tumor volume (**B**) and body weight (**C**) of mice in different groups. (*P ≤ 0.05; **P ≤ 0.01). **D** Immunofluorescence of CD31 (red), HIF-1α (red) and TUNEL (green), and immunohistochemistry of Ki67 and P53 in tumors. Scale bars, 200 μm. **E** H&E staining of heart, liver, spleen, lung and kidney from mice of the four groups. Scale bars, 200 μm. All error bars are expressed as ± SD, n = 6

Encouraged by the efficacy of PAR-Lipo/pEGFP + O_2_@PL in delivering O_2_ and boosting P53 expression in vitro, we aimed to evaluate their antitumor efficacy in MCF-7-induced breast cancer models. When the tumor volume reached 100 to 150 mm^3^, mice were randomly divided into 4 groups (n = 6) and administered according to the scheme in the Fig. 6A. The arrow indicated the corresponding time when the mice received intratumoral injection, and the tumor growth of each group of mice during the whole treatment process was recorded. Significant tumor inhibition was observed in the PAR-Lipo/pTP53 + O_2_@PL and PAR-Lipo/pTP53 groups. Importantly, the PAR-Lipo/pTP5 + O_2_@PL group exhibited a much better therapeutic effect than the PAR-Lipo/pTP5 group without O_2_@PL (P < 0.01). PAR-Lipo/pTP53 alone had limited inhibitory effect on tumor growth, and hypoxia relief could not effectively retarded tumor growth due to the lack of direct tumor killing effect. When the two were combined, a superior synergistic effect was strikingly brought out and broke the treatment bottleneck of p53 gene, the tumor volume in the Saline group at day 20 was up to 14 times that of the initial volume. On the contrary, the combined treatment successfully controlled the final tumor volume below 1.5 times of the initial volume (Fig. 6B). The body weight of the mice showed no obvious changes during treatment (Fig. 6C), indicating the high biological safety of our strategies. It is reported that hypoxia is able to promote the vessel growth in the tumor. Thus, the level of CD31, a ubiquitous blood vessel marker, was observed by immunofluorescence to investigate the angiogenesis after different treatment. To obtain nutrients for growth and to metastasize to distant organs, tumor cells would coopt host vessels, sprout new vessels from existing ones. The resulting vasculature is structurally and functionally abnormal. Blood vessels are leaky, tortuous, dilated, and saccular and have a haphazard pattern of interconnection [34]. As expected, CD31 signal was significantly down-regulated by O_2_@PL, directly supporting the alleviation of hypoxia and the normalization of vascular structure (Fig. 6D). To assess the degree of hypoxic in tumor tissues, the expression of HIF-1α was measured. Similarly, the hypoxic degree of O_2_@PL treated tumor was the lowest. The proliferation of tumor cells after various treatments was analyzed by Ki-67 staining. The number of Ki-67 positive cells in PAR-Lipo/pTP53 + O_2_@PL group treated group was plummeted compared with the other groups, indicating the good tumoral inhibition by O_2_@PL aided PAR-Lipo/pTP53. Cell apoptosis was further measured using TUNEL assay and H&E, the apoptotic cells were the maximum in the combination. The TUNEL results also revealed that more tumor cells were massacred by PAR-Lipo/pTP53, which was consistent with the higher P53 expression. The major tissues of mice in each group were stained by H&E for histopathology analysis. No obvious abnormality was found in all groups compared with the control PBS group (Fig. 6E), suggesting good biocompatibility of the therapeutic procedure. Thus, O_2_@PL could carry oxygen to conquer the hypoxia in tumor, thus enhancing the antitumor ability of gene therapy.

## Discussion

Gene therapy is becoming a promising tool for the treatment of human diseases that cannot be cured by conventional therapies. Hypoxic conditions are energetically challenging for tumor gene therapy since oxygen plays as a limiting factor for transfection efficiency. As one of the most energy-consuming processes in cells, translation requires enormous amounts of ATP, and the production of ATP is extremely oxygen-dependent. Therefore, insufficient oxygen supply results in decreased rate of global mRNA-to-protein translation in the cell due to decreased ATP availability. Gene translation and protein synthesis are blocked under hypoxia, which greatly hinders gene therapy. It was reported that reoxygenation reversed translation in hypoxic cells, which was achieved primarily by the upregulation of oxidative phosphorylation. In addition, acetyl-CoA, the source material of oxidative phosphorylation, can be synthesized from substances other than glucose, so the reoxygenation is still effective even when glucose supply is insufficient.

To solve this problem, we were committed to developing approaches to alleviate tumor hypoxia. Here, we proposed a staged therapeutic strategy. Artificial blood-PFC was chosen as oxygen nanocarrier, and were loaded into liposomes (PFOB@Lipo) to deliver oxygen. PFOB@Lipo were uniform with an average size of about 190 nm, which were stable when stored at 4 ℃. Dissolved oxygen measurements confirmed that the PFOB@Lipo could act as an efficient O_2_ carrier to transport and release oxygen in hypoxia (Fig. 1). We further found that as long as gene expression was performed under normoxia, even if the transfection complexes were added under hypoxia, the transfection efficiency could be greatly restored (Fig. 2). These data provided experimental support for this assumption, that was, hypoxia alleviation could rescue transfection efficiency. In vitro transfection results demonstrated that O_2_@PL effectively increased the expression of GFP in MCF-7 cells under hypoxia (Fig. 3). To further verify the synergism in vivo, mouse orthotopic breast cancer was established. Consistent with the in vitro results, compared with PAR-Lipo/pTP53 group, there was stronger GFP expression in the MCF-7 tumors injected with PAR-Lipo/pTP53 + O_2_@PL (Fig. 4). Based on these data, we chose to deliver O_2_@PL to reverse hypoxia first to reverse hypoxia, and then the therapeutic gene pTP53 was employed. The results showed that O_2_@PL effectively improved the capacity of each transfection complexes to induce tumor cell necrosis and apoptosis, manifested as a greatly increased level of P53 protein while a sharply decreased of HIF-1α protein (Fig. 5). Subsequent in vivo anti-tumor experiments confirmed that O_2_@PL rescued the translation inhibition caused by hypoxia, thereby remarkably promoting the therapeutic effect of P53 and greatly inhibiting the tumor growth (Fig. 6). As the first attempt to use oxygen nanocarrier to enhance gene therapy under hypoxia, our work provided an important guideline for future clinical gene therapy of cancer.

## Conclusion

In summary, the most important factor limiting solid tumor gene therapy is the high dependence of transfection efficiency on oxygen. However, an inevitable nature of the TME is hypoxia, which causes the reduction in intracellular ATP and the inhibition of gene expression simultaneously. We demonstrated that the topical delivery of oxygen alleviated tumor hypoxia. Then, the endoplasmic reticulum targeting cationic liposome PAR-Lipo efficiently delivered the tumor suppressor gene pTP53, leading to increased expression of P53 in tumors to inhibit tumor growth. This work provided a simple but effective strategy to rescue the poor gene therapy on malignant hypoxic tumors.

## Experimental section

### Reagents

Egg phosphatidyl lipid-80 (E80) and cholesterol were obtained from lipoid Co. (Ludwigshafen, Germany). 1,2-Dioleoyl-3-trimethylammoniumpropane (DOTAP) was purchased from Avanti Co. Ltd. (USA). 1,2-Dioleoyl-sn-glycero-3-phosphoethanolamine (DOPE) and distearoyl-sn-glycero-3-phosphoethanolamine-N- [maleimide (polyethylene glycol)-2000] (DSPE-PEG_2000_) were purchased from AVT Co. Ltd. (Shanghai, China). A pardaxin peptide (HGFFALIPKIISSPLFKTLLSAVGGSAVGSALSSGGQE) was synthesized by Qiang Yao Biotech Co. Ltd. (Shanghai, China). MTT reagent, cell cycle and apoptosis analysis kits, BCA protein assay kit, RIPA lysis buffer and protease inhibitor cocktail were acquired from Biotechnology (Jiangsu, China). HRP-conjugated goat anti-rabbit secondary antibody (Proteintech, SA00001-2) and β-actinantibody (Proteintech, 20,536–1-AP) were from Proteintech Group, Inc. (Chicago, USA). Perfluorocarbon (PFOB, B122295) were purchased from Aladdin (Shanghai, China). Lipofectamine 2000 (Lipo 2000) was purchased from Invitrogen (Carlsbad, CA, USA). P53 polyclonal antibody were purchased from Proteintech Group, Inc. (Wuhan, China). Hypoxia-inducible factor 1-alpha (HIF-1α) antibody (ab179483) were from Abcam (Abcam, USA). RPMI 1640 medium (RPMI), Dulbecco's modified Eagle's medium (DMEM) and penicillin/streptomycin (100 U/mL) were obtained from JiNuo Biotechnology Co. Ltd. (Zhejiang, China). Enhanced green fluorescent protein (EGFP)-encoding plasmid DNA (pEGFP) was kindly provided by the First Affiliated Hospital College of Medicine, Zhejiang University (Hangzhou, China). The P53 plasmid (pTP53) was synthesized by Youbao Technology Biological Co. Ltd. (Changsha, China). All plasmids were amplified by Weizhen Biotech Co. Ltd. All the chemicals and solvents were of analytical grade.

### Cell lines

Human breast cancer cells (MCF-7) were obtained from the Institute of Biochemistry and Biology (Shanghai, China) and were cultured in Dulbecco's Modified Eagle Medium (DMEM) medium containing 10% fetal bovine serum (FBS) at 37 °C in a humidified atmosphere with 5% CO_2_. Hypoxia was generated using a hypoxia incubator (Eppendorf Galaxy® 48 R, Germany) at 2% O_2_, 5% CO_2_ and 93% N_2_.

### Animals

Female BALB/c nude mice (18–22 g, 5–8 week) were purchased from Slaccas Experimental Animal Co. Ltd. (Shanghai, China) and were housed in appropriate animal facilities at Zhejiang University.

### Preparation and characterization of liposomes

A lipid film hydration-sonication method was employed to prepare liposomes containing PFOB (PFOB@Lipo). Briefly, 20 mg E80, 5 mg cholesterol, 20ug DSPE-PEG_2000_ and PFOB (60 μL) were dissolved in 5 mL chloroform, evaporated at 45 °C to form a thin lipid film and hydrated with 2 mL PBS buffer (pH = 7.4). The suspensions were stored in the refrigerator (4 °C) overnight before use. The morphology and size of the nanoparticles was determined by transmission electron microscopy (TEM) (JEM-1230, Japan) and dynamic light scattering (DLS) (Malvern Nano-ZS 90, Malvern, UK), respectively.

### Measurement of O2 Loading Capacity of PFOB@Lipo

Firstly, the deoxygenated culture medium (5 mL) was added into a three -necked flask (25 mL) which is sealed by rubber plugs. The oxygen concentration of the solution was measured by the probe of portable dissolved oxygen meter (Rex, JPF-605B, China), which is inserted through rubber plug into the flask. Subsequently, 5 mL oxygen-saturated PBS, O_2_@PL and PFOB solutions (60 μL PFOB/mL) were injected via syringe into the closed flask and then the oxygen concentration was recorded at the time of oxygen equilibrium.

### Cytotoxicity of the liposomes

The cytotoxicity of PFOB@Lipo were assessed in MCF-7 cells using the MTT method according to the manufacturer's suggested procedures. The cells were incubated with various liposomes for 48 h under normoxia and hypoxia. The data were presented as the percentage of surviving cells and represent the mean values of 5 measurements.

### Cellular internalization

The MCF-7 cells were observed with DiD-labeled PAR-Lipo (10 μg/mL) for 2, 6, or 8 h. The transfeciton complexes were obtained by mixing PAR-Lipo (14 μg) with Cy3-labeled DNA (0.5 μg) at 37 °C for 30 min. MCF-7 cells were incubated with the complexes for different periods of time under normoxia (20% O_2_) or hypoxia (2% O_2_). Then, the cells were rinsed with PBS, stained with DAPI, and observed by a fluorescence microscopy (Nikon, Japan).

### qRT-PCR

Total RNA from MCF-7 cells was extracted with TRIzol RNA isolation reagents (Servicebio), followed by cDNA synthesis with the High-Capacity cDNA Reverse Transcription Kit (Thermo). qRT-PCR was conducted with FastStart Universal SYBR Green Master (Rox) (Servicebio).

### In vivo transfection

In situ MCF-7 tumor models were established by subcutaneous inoculation of 1 × 10^7^ cells into the right pads of female BALB/c nude mice. Mice were peritumorally injected with 50 μL O_2_@PL (60 μL PFOB/mL) for two days, and then peritumorally injected with complexes of PAR-Lipo/pEGFP (25.0 μg/mL pDNA and 0.125 mg/mL PAR-Lipo), with the same dosage of saline, O_2_@PL and complexes of PAR-Lipo/pEGFP as controls. As a comparison, the control groups consisting of saline, O_2_@PL and complexes of PAR-Lipo/pEGFP alone at the same concentration of the experimental group were conducted as well. The nude mice were anaesthetized at desired time interval (48 h) to detect the fluorescent signals of EGFP. Then, they were sacrificed with their major organs collected for ex vivo imaging.

### In vitro transfection

The MCF-7 cells were inducted with Lipo 2000/pEGFP, PEI/pEGFP or PAR Lipo/pEGFP complexes containing pEGFP at a final concentration of 3.2 μg/mL for 5 h at 37 °C. After refreshed cell culture medium, the cells were treated with 50 μL O_2_@PL (60 μL PFOB/mL) under normoxia or hypoxia for 24 h. Then, the gene transfection efficiency was examined by fluorescence microscopy. The images were acquired with constant parameters for different groups. Positive rate of GFP expression was determined by flow cytometry after 24 h transfection.

### Cell killing effect in vitro

pTP53 was selected as therapeutic genes. MCF-7 cells were incubated with the complexes of PAR-Lipo/pTP53 (5:1, w/w), PEI/pTP53 (5:1, w/w), Lipo 2000/pTP53 (1.5:1, w/w). After 5 h of incubation, the medium was replaced with complete culture medium. After 48 h of transfection with or without 50 μL O_2_@PL (60 μL PFOB/mL), the apoptosis/necrosis rate and protein expression were measured by flow cytometry.

For the western blotting, cell lysates were prepared by a RIPA lysis buffer containing protease inhibitors.The total protein was determined with the BCA protein assay kit. Equal amounts of protein were electrophoresed in a 12% SDS-PAGE bis–tris gel (Invitrogen, Carlsbad, CA) and then transferred onto nitrocellulose membranes (Da Wen Biotechnology Co. Ltd., Hangzhou, China). The membranes were blocked with 5% skimmed milk for 2 h at room temperature and incubated overnight with rabbit anti-mouse P53 antibody (1:1000) and rabbit anti-mouse HIF-1α antibody (1:1000) at 4 °C. The protein bands were incubated with HRP-conjugated goat anti-rabbit antibodies (1:10,000) for 2 h and were subsequently visualized using the ECL Plus kit (Beyotime Institute of Biotechnology. Jiangsu, China).

### Anticancer efficacy in vivo

MCF-7 tumor-bearing mice were randomly divided into four groups (n = 6). Mice in group 2 and 4 were peritumorally injected with 50 μL O_2_@PL (60 μL PFOB/mL) every three days. The mice in group 3 and 4 were peritumorally injected with PAR-Lipo/pP53 (5:1, w/w) at a plasmid dose of 0.25 mg/kg, once every 3 days, 7 times in total. Tumor size and body weight of mice were measured every other day, and the tumor volume was calculated using the formula (tumor volume = length × width × height/2). At the end of the experiments, the tumors and major organs were collected, embedded in paraffin, stained with hematoxylin and eosin (H&E) and immune-stained for HIF-1α, TUNEL, CD31, Ki-67 and P53. Representative images were collected using a microscope (Nikon, Japan).

### Semi-quantification by ImageJ

For each experiment, three or more fields of view were taken as fluorescence images for each group. Then, the fluorescence intensity of each image was semi-quantitated with ImageJ software, and the values were averaged.

### Statistical analysis

Quantitative data are expressed as the mean ± S.D. When only two groups were compared, paired t test was used. And an evaluation of significance was performed using a one-way ANOVA when more than two groups were compared. All statistical analyses were conducted using GraphPad Prism 8 software. P values less than 0.01 were considered statistically significant.

## Data Availability

The data and materials of the study can be obtained from the corresponding author upon request.
